# Anti-inflammatory effects of a traditional Korean medicine: Ojayeonjonghwan

**DOI:** 10.1080/13880209.2017.1339282

**Published:** 2017-06-14

**Authors:** Sun-Young Nam, Kyu-Yeob Kim, Mi Hye Kim, Jae-Bum Jang, So-Young Rah, Jin-Man Lee, Hyung-Min Kim, Hyun-Ja Jeong

**Affiliations:** a Department of Pharmacology, College of Korean Medicine, Kyung Hee University, Seoul, Republic of Korea;; b Department of Food and Nutrition, Hoseo University, Asan, Chungnam, Republic of Korea;; c Department of Pharmaceutical Engineering, Hoseo University, Asan, Chungnam, Republic of Korea;; d Department of Biochemistry, Chonbuk National University, Jeonju, Republic of Korea;; e Department of Food Science & Technology and Research Institute for Basic Science, Hoseo University, Asan, Chungnam,Republic of Korea

**Keywords:** Mouse peritoneal macrophages, nitric oxide, inflammatory cytokine, NF-κB

## Abstract

**Objective:** To study the anti-inflammatory properties of OJ.

**Context:** Ojayeonjonghwan (OJ) is a traditional Korean prescription, which has been widely used for the treatment of prostatitis. However, no scientific study has been performed of the anti-inflammatory effects of OJ.

**Materials and methods:** Peritoneal macrophages were isolated 3–4 days after injecting a C57BL/6J mouse with thioglycollate. They were then treated with OJ water extract (0.01, 0.1, and 1 mg/mL) for 1 h and stimulated with lipopolysaccharide (LPS) for different times. Nitric oxide (NO), inducible nitric oxide synthase (iNOS) and cyclooxygenase (COX)-2, and proinflammatory cytokine levels were determined by NO assay, Western blotting, RT-PCR and ELISA.

**Results:** NO generation and iNOS induction were increased in the LPS-activated mouse peritoneal macrophages. However, NO generation and iNOS induction by LPS were suppressed by treatment with OJ for the first time. The IC_50_ value of OJ with respect to NO production was 0.09 mg/mL. OJ did not influence LPS-stimulated COX-2 induction, but did significantly decrease LPS-stimulated secretions and mRNA expressions of tumour necrosis factor (TNF)-α, interleukin (IL)-6, and IL-1β. Inhibition rates of TNF-α, IL-6, and IL-1β at an OJ concentration of 1 mg/mL were 77%, 88%, and 50%, respectively. OJ also suppressed the LPS-induced nuclear translocation of NF-κB. High-performance liquid chromatography showed schizandrin and gomisin A are major components of OJ.

**Conclusions:** OJ reduces inflammatory response, and this probably explains its positive impact on the prostatitis associated inflammation.

## Introduction

Chronic inflammation leads to the progression of immune diseases, such as prostatitis, inflammatory bowel disease, gastritis, rheumatoid arthritis, cancer, and atherosclerosis (Hanahan and Weinberg 2000; Palapattu et al. 2004). Prostatitis is an asymptomatic inflammation of the prostate and results from identifiable prostatic infections. The aetiology of prostatitis is unknown, but proinflammatory cytokines have been detected in secreted prostatic fluid (Alexander et al. 1998). Excessive levels of tumour necrosis factor (TNF)-α and interleukin (IL)-1β have been detected in seminal plasma of patients with prostatitis (Alexander et al. 1998; Orhan et al. 2001). Nadler et al. (2000) similarly showed that the increased levels of proinflammatory cytokines are closely linked to prostatic inflammation. Furthermore, certain cytokines play important roles in nitric oxide (NO) generation in cells under normal and urothelial tumour conditions (Hosseini et al. 2006), and thus, levels of proinflammatory mediators might be of predictive value in prostatitis.

Macrophages are responsible for innate immune response. They secrete inflammatory mediators by recognizing and phagocytosing pathogens, and regulate acquired immunity (Beurel and Jope 2009). Macrophage activation promotes inflammatory processes (Nishimura et al. 1991; Medzhitov and Janeway 1997a; Chou et al. 2014), and NO generation and pro-inflammatory cytokine levels, including those of IL-6, IL-1β, and TNF-α, which induce the recruitment of other inflammatory cells (Medzhitov and Janeway 1997b; Gantt et al. 2001). Macrophages also increase inflammatory reactions by inducing nitric oxide synthase (iNOS) and cyclooxygenase (COX)-2 (Kim et al. 2008).

The pathogenesis and aetiology of nonbacterial prostatitis is incompletely understood. Despite various treatment modalities, it is difficult to cure nonbacterial prostatitis, which promotes research directed at the developments of novel drugs that are effective and free of side effects. Traditional Korean medicine provides an excellent resource for biomaterials containing secondary metabolites that exhibit anti-inflammatory activities (Burk et al. 2009). Ojayeonjonghwan (OJ, liquid dosage form) is a traditional Korean prescription that has been widely used for the treatment of prostatitis. OJ is composed of *Schisandra chinensis* Baillon (Schizandraceae), *Lycium barbarum* Linnaeus (Solanaceae)*, Cuscuta chinensis* Lamark (Convolvulaceae)*, Rubus coreanum* Miquel (Rosaceae), and *Plantago asiatica* Linne (Plantaginaceae), which are also used to treat male sexual dysfunction. Traditionally, *Schisandra chinensis* has been used to treat kidney disease, the common cold, and memory deficiencies. Furthermore, it has been reported *Schisandra chinensis* exhibits anti-inflammatory effects in propionibacterium acnes-stimulated THP-1 and UVB-irradiated human fibroblasts HDF cells (Guo et al. 2016). Extracts of *Lycium barbarum* were found to exhibit anti-inflammatory activities against carrageenan induced rat paw oedema and CCl_4_-induced liver injury (Lin et al. 1997), and *Cuscuta chinensis* exhibited an antioxidant effect in human sperm (Yang et al. 2006). However, the pharmacological mechanisms responsible for the therapeutic effects of OJ on prostatitis have not been determined. We assumed that OJ might be helpful for the treatment of chronic prostatitis. Therefore, in the present study, we investigated the effects of OJ on lipopolysaccharide (LPS)-stimulated NO generation, on the induction of iNOS and COX-2 in mouse peritoneal macrophages, and on inflammatory cytokine secretion and nuclear factor-κB (NF-κB) regulation.

## Materials and methods

### Reagents

Dulbecco’s modified Eagle medium (DMEM), LPS, gomisin A (GA, purity 98%), and 3-(4, 5-dimethylthiazol-2-yl)-2,5-diphenyltetrazolium bromide (MTT) were purchased from Sigma (St. Louis, MO). Anti-mouse TNF-α (551225), biotinylated anti-mouse TNF-α (554415), recombinant mouse TNF-α (554589), anti-mouse IL-6 (554400), biotinylated anti-mouse IL-6 (554402), and recombinant mouse IL-6 (554582) were obtained from Pharmingen (San Diego, CA). Antibodies for iNOS, COX-2 (SC-1745), NF-κB (SC-7151), phosphorylated (p)IκB-α (SC-8404), and glyceraldehyde-3-phosphate dehydrogenase (GAPDH; SC-32233) were obtained from Santa Cruz Biotechnology, Inc. (Santa Cruz, CA). Thioglycollate (TG) was purchased from Difco Laboratories (Detroit, MI), and anti-mouse IL-1β (MAB 401), biotinylated anti-mouse IL-1β (BAF 401), and recombinant mouse IL-1β (401-ML) were purchased from R&D Systems (Minneapolis, MN). Foetal bovine serum (FBS) was purchased from Life Technologies (Grand Island, NY). Schizandrin (purity 99%) was purchased from Wako Pure Chemical (Osaka, Japan).

### Preparation of OJ

A sample of OJ (Table 1) was obtained from an oriental drug store, Noa Pharmacy (Seoul, Republic of Korea), and authenticated by Kim HM, College of Korean Medicine, Kyung Hee University. A voucher specimen was deposited at the College of Korean Medicine, Kyung Hee University. OJ was extracted by decocting dried herbs with boiling distilled water for approximately 2 h. The decoction was then filtered, lyophilized, and kept at 4 °C. Dilutions were made with distilled water, and the finally extract was filtered through a 0.22 μm syringe filter.

**Table. 1.** Components of Ojayeonjonghwan (OJ)

Scientific name	Dose (g)
*Lycium barbarum* Linne	9
*Cuscuta chinensis* Lamark	7
*Rubus coreanum* Miquel	5
*Plantago asiatica* Linne	3
*Schisandra chinensis* Baillon	1
Total	25

### Animals

The original stock of male C57BL/6J mice was purchased from the Dae-Han Experimental Animal Center (Eumseong, Republic of Korea). Animals were housed at 23 ± 1 °C under a 12/12-h light–dark cycle. Food and water were provided *ad libitum*. All protocols were approved by the institutional animal care and use committee of Kyung Hee University.

### Peritoneal macrophages culture

Macrophages were harvested 3–4 days after intraperitoneally injecting 2.5 mL TG into mice and then isolated, as reported previously (Xie et al. 1994). Briefly, peritoneal lavage was conducted using 8 mL of DMEM. Next, the cells were distributed into 24-well tissue culture plates (5 × 10^5^ cells/well) in DMEM supplemented with 10% heat-inactivated FBS. Cells were then incubated for 3 h at 37 °C in a 5% CO_2_ atmosphere, washed 3 times with DMEM to remove non-adherent cells, and equilibrated with DMEM containing 10% FBS.

### MTT assay

Cell viability was determined using an MTT assay. Briefly, 500 μL aliquots of a peritoneal macrophage cell suspension (5 × 10^5^ cells/well) were cultured in 24-well plates for 24 h after being treated with different concentrations of OJ. Fifty microlitre of MTT solution (5 mg/mL) was then added to the plates and incubated for 4 h at 37 °C. Next, the supernatant was removed and the insoluble formazan product was dissolved in dimethyl sulphoxide. The optical density of the culture plates at 540 nm was then measured by using an enzyme-linked immunosorbent assay (ELISA) reader. The optical density of formazan formed in untreated control cells was taken to be 100% viability.

### Measurement of NO concentration

Peritoneal macrophages (3 × 10^5^ cells/well) were pretreated with OJ (0.01, 0.1, and 1 mg/mL) for 1 h and then stimulated with LPS (1 μg/mL) for 48 h. To measure the nitrite concentration, 100 μL aliquots were removed from conditioned medium and incubated with an equal volume of Griess reagent (1% sulphanilamide/0.1% *N*-(1-naphtyl)-ethylenediamine dihydrochloride/2.5% H_3_PO_4_) at room temperature for 10 min. The absorbance at 540 nm was then determined by using ELISA reader. The NO_2_^−^ concentration was determined by using sodium nitrite as a standard. This value was determined for each experiment and then subtracted from the value obtained from medium that contained peritoneal macrophages.

### Reverse transcription polymerase chain reaction (RT-PCR) analysis

Total RNA was isolated from mouse peritoneal macrophages using an easy-BLUE mRNA extraction kit (17061; iNtRON Biotech, Kyunggi-do, Korea). The concentration of total RNA in final eluate was determined by spectrophotometry. Total RNA (2.0 μg) was heated at 65 °C for 10 min and then chilled on ice. cDNA was reverse-transcribed using a cDNA synthesis kit (K-2041; iNtRON Biotech, Kyunggi-do, Korea) for 90 min at 37 °C. PCR was performed using the following primers: COX-2 (5′-GGA GAG ACT ATC AAG ATA GTG ATC-3′; 5′-ATG GTC AGT AGA CTT TTA CAG CTA-3′), iNOS (5′-TCA CTG GGA CAG CAC AGA AT-3′; 5′-TGT GTC TGC AGA TGT GCT GA-3′), TNF-α (5′-ATG AGA ACA GAA AGC ATG ATC-3′; 5′-TAC AGG CTT GTC ACT CGA ATT-3′), IL-6 (5′-CGG GAT CCA TGT TCC CTA CTT CAC AA-3′; 5′'-CCC AAG CTT CTA CGT TTG CCG AGT AGA-3′), IL-1β (5′-AGG CCA CAG GTA TTT TGT CG-3′; 5′-GCC CAT CCT CTG TGA CTC AT-3′), and GAPDH (5′-GGC ATG GAC TGT GGT CAT GA-3′; 5′-TTC ACC ACC ATG GAG AAG GC-3′) which was used as an internal control to confirm equal amounts of RNA were subjected to reverse transcription. Annealing temperatures were 60 °C for COX-2, 58 °C for iNOS and TNF-α, 50 °C for IL-1β, and 62 °C for IL-6 and GAPDH. The products were electrophoresed on 1.5% agarose gels and visualized by ethidium bromide staining.

### Preparation of nuclear and cytosolic extracts

Briefly, after activating cells for the indicated times, they were washed in ice-cold phosphate-buffered saline and centrifuged at 15,000 *g* for 1 min. Cells were then resuspended in 40 μL of cold hypotonic buffer (10 mM HEPES/KOH, 2 mM MgCl2, 0.1 mM EDTA, 10 mM KCl, 1 mM DTT, and 0.5 mM PMSF, pH 7.9) and allowed to swell on ice for 15 min. Cells were then lysed with 2.5 μL of 10% Nonidet P (NP)-40, and centrifuged at 15,000 *g* for 3 min at 4 °C. Supernatants (cytosolic protein) and pellets were resuspended in 40 μL of cold saline buffer (50 mM HEPES/KOH, 50 mM KCl, 300 mM NaCl, 0.1 mM EDTA, 10% glycerol, 1 mM DTT, and 0.5 mM PMSF pH 7.9) and left on ice for 20 min. After centrifugation (15,000 *g* for 15 min at 4 °C), aliquots of supernatants containing nuclear proteins were frozen in liquid nitrogen and stored at −80 °C until required for analysis. A bicinchoninic acid protein assay (Sigma, St. Louis, MO) was used to measure protein concentrations.

### Western blot analysis

Stimulated peritoneal macrophages were lysed and separated by 12% SDS-PAGE. After electrophoresis, proteins were transferred to nitrocellulose membranes, which were then blocked for 2 h with 1 × phosphate-buffered saline Tween-20 (PBST) containing 5% skim milk. After treatment with primary Ab (1:500 in PBST) by incubation overnight at 4 °C, membranes were washed for 5 × 15 min with PBST. To detect proteins, blots were incubated with secondary Ab (1:3000 in PBST) conjugated with peroxidase for 40 min. Finally, protein bands were visualized by enhanced chemiluminescence (Amersham Co., Newark, NJ) according to the manufacturer’s instructions.

### Cytokines assay

TNF-α, IL-6, and IL-1β secretions were measured by ELISA, as described previously (Kim et al. 2014). Briefly, 24-well plates were coated with 100 μL aliquots of anti-mouse TNF-α, IL-6, and IL-1β (1.0 μg/mL), respectively, and incubated overnight at 4 °C. After washing with PBST, 100 μL of cell medium or TNF-α, IL-6, and IL-1β standards was added and incubated at 37 °C for 2 h. After washing wells, biotinylated anti-mouse TNF-α, IL-6, or IL-1β (0.2 μg/mL) was added and incubated at 37 °C for an additional 2 h. Wells were washed, avidin–peroxidase was added and incubated for 30 min at 37 °C and then wells were then rewashed, and 2, 2″azino-bis (3-ethylbenzthiazoline-6-sulphonic acid) substrate was added. Colour development at 405 nm was measured by using an automated microplate ELISA reader. Standard curve was generated for each assay plates by measuring the absorbencies of serial dilutions of recombinant TNF-α, IL-6, and IL-1β at 405 nm.

### High-performance liquid chromatography (HPLC)

OJ extract, schizandrin, or GA was filtered through a 0.2 μm membrane, and 20 μL aliquots of filtrates were subjected to HPLC. A Syncronis C18 (150 mm × 2.1 mm; particle size 5 μm) analytical column was used. The mobile phase was a 70:30 mixture of solvent A (methanol) and solvent B (DI water). The analysis was carried out at a flow rate of 0.6 mL/min, and the injection volume was 20 μL. HPLC runs took ∼55 min.

### Statistical analysis

Results are presented as the means ± standard errors of means (SEMs) of at least three different experiments. The analysis was conducted using the independent *t*-tests and ANOVA with Tukey’s *post hoc* test. Statistical significance was accepted for *p* values <0.05.

## Results

### OJ inhibited NO generation and iNOS induction

**Figure 1.**
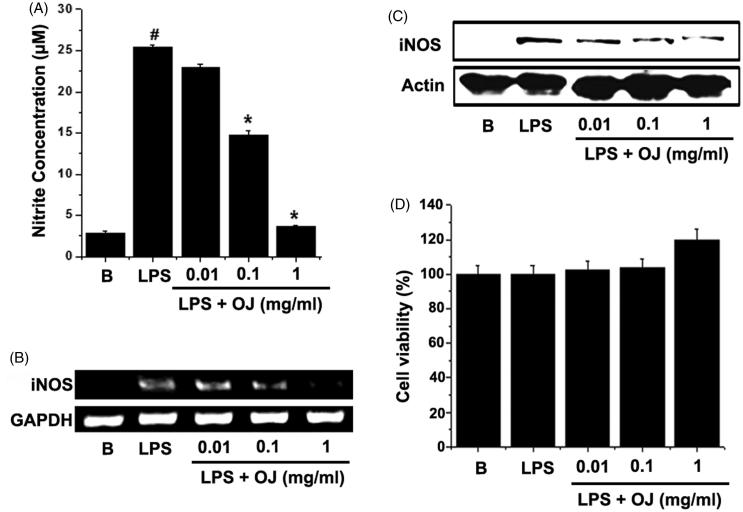
OJ inhibited NO generation and iNOS induction. Cells (5 × 10^5^ cells/well) were treated with OJ for 1 h and then treated with LPS (1 μg/mL) for 48 h. Nitrite concentrations were measured in cell supernatants using the Griess method (A). Cells (5 × 10^6^ cells/well) were treated with OJ for 1 h and then treated with LPS (1 μg/mL) for 24 h. The mRNA and protein expressions of iNOS were measured by RT-PCR (B) and Western blot analysis (C). Cell viability was determined using a MTT assay (D). #*p <* 0.05, significantly different from non-treated cells. ∗*p <* 0.05, significantly different from LPS-stimulated cells. B: non-treated cells.

In order to examine the effects of OJ on LPS-stimulated NO generation, peritoneal macrophages were incubated with different doses of OJ for 1 h. Thereafter, cells were stimulated with LPS for 48 h. The resultant NO levels were then measured. OJ significantly and dose dependently inhibited LPS-induced NO generation (Figure 1(A)), and thus, iNOS induction was analyzed in OJ-treated cells. LPS significantly up-regulated iNOS mRNA and protein, whereas pretreatment with OJ dose dependently suppressed these up-regulations (Figure 1(B,C)). The cell viabilities were studied using an MTT assay, and OJ was not found to have any toxic effect (Figure 1(D)).

### Effect of OJ on COX-2 induction

**Figure 2.**
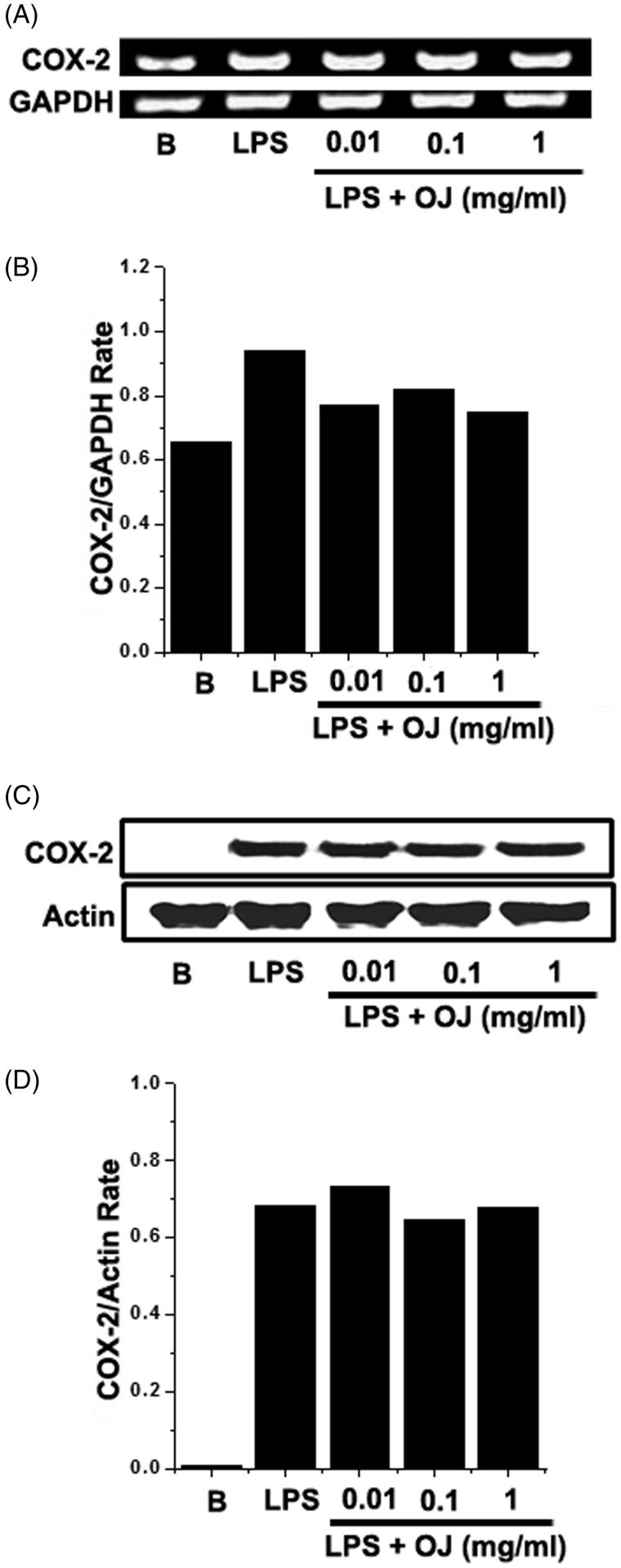
Effect of OJ on the COX-2 induction. Cells (5 × 10^6^ cells/well) were treated with OJ for 1 h and then treated with LPS (1 μg/mL). The mRNA and protein expressions of COX-2 were measured by using RT-PCR (A) and Western blot analysis (C). Relative mRNA (B) and protein (D) levels were quantitated by densitometry. B: non-treated cells.

Western blot analysis and RT-PCR were performed to examine the effects of OJ on LPS-stimulated COX-2 induction in mouse peritoneal macrophages. Peritoneal macrophages were incubated with OJ at the different concentrations. Pretreatment with OJ was not found to significantly inhibit in the inductions of COX-2 mRNA or protein (Figure 2).

### OJ suppressed TNF-α, IL-6, and IL-1β production and mRNA induction

**Figure 3.**
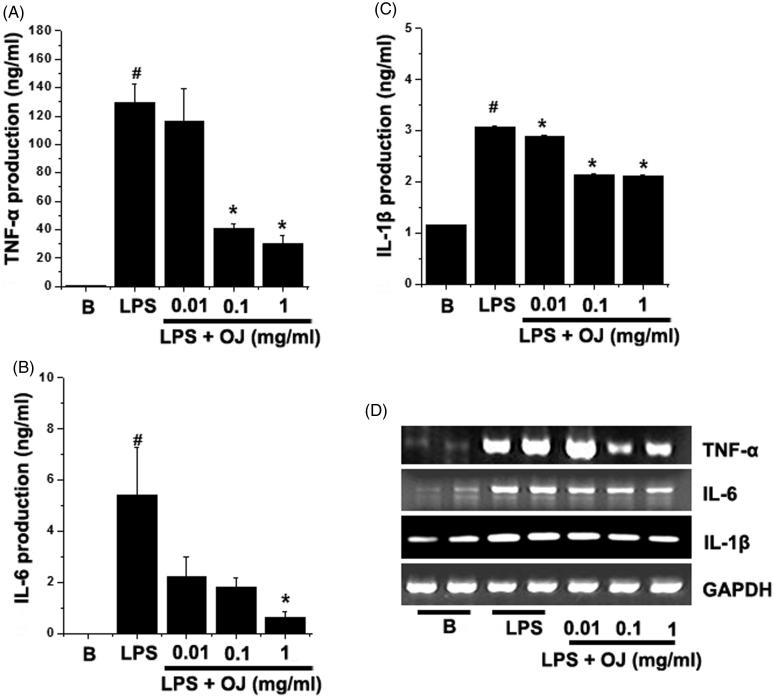
OJ suppressed TNF-α, IL-6, and IL-1β production and mRNA induction. Cells (5 × 10^5^ cells/well) were treated with OJ (0.01, 0.1, and 1 mg/mL) for 1 h and then treated with LPS (1 μg/mL) for 24 h. The productions of TNF-α (A), IL-6 (B), and IL-1β (C) in cell supernatants were measured by ELISA. The mRNA expression was measured by RT-PCR (D). Results are presented as the means ± SEM of three independent experiments. #*p <* 0.05, significantly different from non-treated cells. ∗*p <* 0.05, significantly different from LPS-stimulated cells. B: non-treated cells.

ELISA was used to study the effects of OJ on LPS-stimulated TNF-α, IL-6, and IL-1β secretion. Peritoneal macrophages were incubated with OJ for 1 h and then treated with LPS for 24 h. Figure 3 showed that the productions of TNF-α, IL-6, and IL-1β were dramatically increased by LPS. When OJ was pretreated, it dose dependently inhibited the LPS-induced productions of TNF-α and IL-6 (Figure 3(A,B). In fact, OJ significantly suppressed the IL-1β production in all groups (Figure 3(C)). To determine whether OJ regulates the LPS-stimulated mRNA levels of these cytokines and we next performed RT-PCR. LPS massively induced TNF-α, IL-6, and IL-1β mRNA, and these increases were markedly suppressed by OJ pretreatment (Figure 3(D)).

### OJ inhibited activation of NF-κB

**Figure 4.**
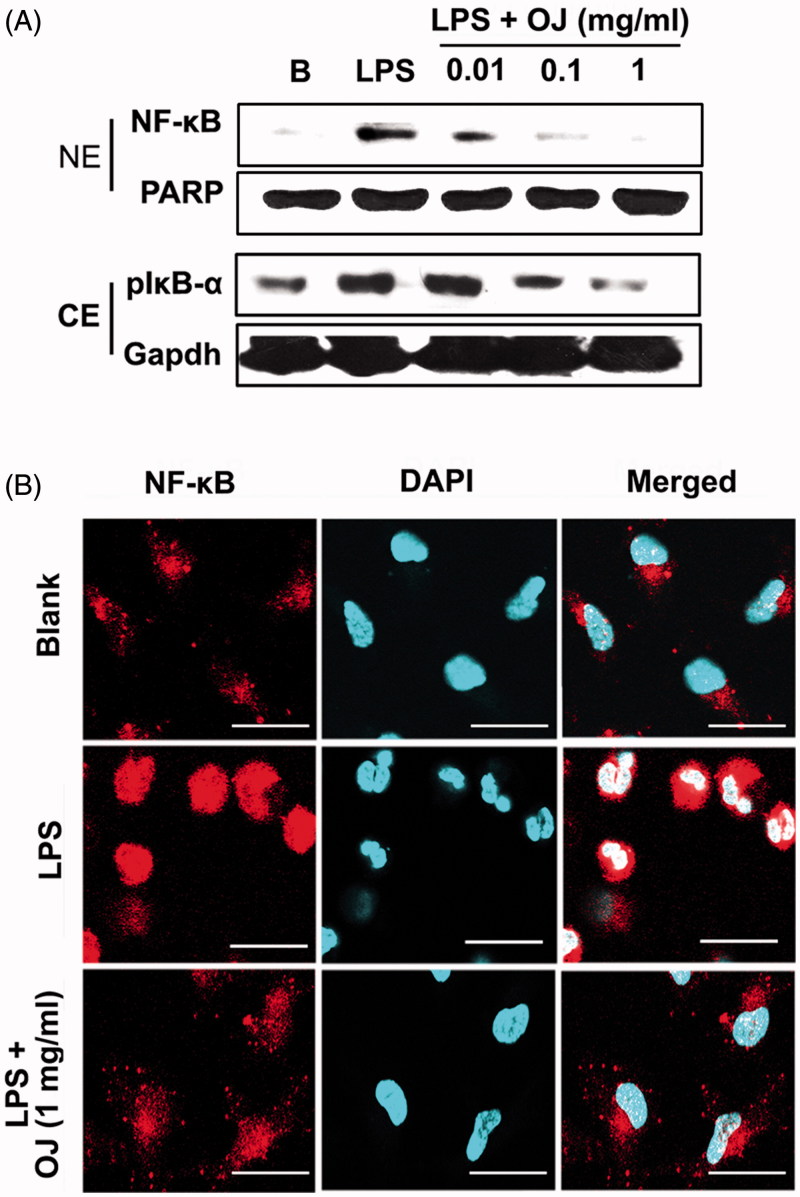
OJ inhibited activation of NF-κB. Cells (5 × 10^6^ cells/well) were treated with OJ (0.01, 0.1, and 1 mg/mL) for 1 h and then stimulated by LPS (1 μg/mL) for 2 h. NF-κB and phosphorylated IκB-α were determined by Western blot analysis (A). NF-κB was stained using a primary anti-p65 for 1 h and then incubated with secondary TRITC-conjugated IgG for 1 h (B). B: non-treated cells. NE: nuclear extract; CE: cytoplasmic extract; PARP: poly(ADP-ribose) polymerase-1.

OJ suppressed LPS-stimulated NF-κB activation in peritoneal macrophages (Figure 4(A), upper panel), and Western blotting showed OJ suppressed LPS-stimulated IκB-α phosphorylation (a marker of NF-κB activation) in cytoplasmic lysates (Figure 4(A), lower panel). Furthermore, immunocytochemistry showed OJ pretreatment inhibited the LPS-induced nuclear translocation of NF-κB (Figure 4(B)).

### HPLC analysis of OJ and its compounds

**Figure 5.**
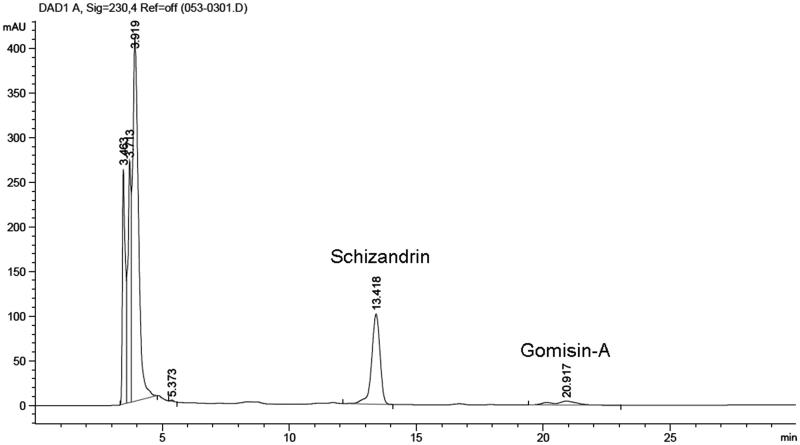
HPLC chromatogram of OJ. The chromatogram was obtained by monitoring absorbance at 230 nm.

Preliminary qualitative phytochemical screening of OJ extract was performed to identify bioactive components. Schizandrin and GA were identified by HPLC using standard controls at retention times of ∼13 and 20 min, respectively (Figure 5). Schizandrin and GA in OJ were included about 16.8% and 2.1%, respectively.

## Discussion

We found that OJ inhibited the LPS-stimulated NO and pro-inflammatory cytokine induction in mouse peritoneal macrophages, and the LPS-induced iNOS and proinflammatory cytokine expression and NF-κB activation.

NO is a key inflammatory mediator and generated by iNOS under various physiological and pathological conditions. Excessive levels of NO are able to cause cell death, reactive oxygen species accumulation, and disrupt tissue homeostasis (Pando and Verma 2000). NO was used as a marker of inflammation in patients with prostatitis syndrome (Hosseini et al. 2006). COX-2 is also increased during inflammation and is markedly expressed in activated macrophages and monocytes (Meade et al. 1993; Crofford et al. 2000). Furthermore, macrophages stimulated by LPS secret TNF-α, IL-6, and IL-1β (Medzhitov and Janeway 1997b). IL-1 is also known as lymphocyte-activating factor (Masihi et al. 1997) and is produced by diverse cell types, especially macrophages. IL-1 stimulates lymphocytes and acts as a mediator of immune response (Dinarello 1989). On the other hand, IL-6 is a produced by a variety of immune cells, such as monocytes and macrophages (Van Snick 1990). TNF-α leads to leukocyte infiltration, inflammation, tissue fibrosis, and granuloma formation and supports inflammatory responses by inducing inflammatory cytokine secretion (Baumgartner et al. 1994). Together, these inflammatory mediators have been implicated in prostatitis. Nishimura et al. (1991) reported that macrophages were stimulated in prostatitis, and thus, the suppression of macrophage activation is positively associated with the inhibition of prostatitis. In the present study, OJ suppressed the LPS-induced secretions of NO, TNF-α, IL-6, and IL-1β. These results suggest that OJ has anti-inflammatory activities in prostatitis.

NF-κB is necessary for the induction of inflammatory mediator secretion and the induction of iNOS and COX-2 (Libermann and Baltimore 1990; Xie et al. 1994; Oh et al. 2012). In cytoplasm, NF-κB is bound to inhibitory molecule, IκB (Sen and Baltimore 1986), and IκB is degraded and phosphorylated by diverse extracellular stimuli. Liberated NF-κB then translocated to the nucleus and binds to the κB motifs in the promoter regions of various genes, including those of proinflammatory mediators (Lenardo and Baltimore 1989). Notably, in the present study, OJ effectively suppressed the nuclear translocation of NF-κB. Although NF-κB activation was inhibited by OJ, it did not change COX-2 expression. Additional studies are required to determine the effect of OJ on COX-2 pathways mediated by macrophages.

As described above, OJ consists of five different herbs. *Schisandra chinensis* has anticancer, antioxidative, antihepatotoxic, anti-HIV, and anti-inflammatory properties (Liu 1989; Yasukawa et al. 1992). *Schisandra chinensis* fruits contain pharmacologically active lignans such as GA and lignans with a dibenzocyclooctadiene skeleton (Kuo et al. 1997; Chen et al. 1998; Choi et al. 2006). In a previous study, Schizandrae-derived lignans were found to have anti-inflammatory properties and to promote HL-60 cell apoptosis (Chen et al. 1999). It has also been reported schizandrin inhibits prostaglandin E_2_ release, NO production, and the inductions of COX-2 and iNOS by inhibiting NF-κB activation (Guo et al. 2008). Ohkura et al. (1999) reported that GA has anti-inflammatory effects, and in our previous study, we observed GA strongly inhibited inflammatory reactions in LPS-induced macrophages (Jeong et al. 2014). In addition, OJ contains schizandrin and GA as active components.

OJ suppressed the LPS-induced iNOS expression and pro-inflammatory cytokine secretion by mouse peritoneal macrophages. Our data provide evidence that OJ plays a role in prostatitis treatment.
